# Preparation and characterisation of PHT-loaded chitosan lecithin nanoparticles for intranasal drug delivery to the brain†

**DOI:** 10.1039/d0ra04890a

**Published:** 2020-08-05

**Authors:** Amal Yousfan, Noelia Rubio, Abdul Hakim Natouf, Aamal Daher, Nedal Al-Kafry, Kerrie Venner, Houmam Kafa

**Affiliations:** Department of Pharmaceutics and Pharmaceutical Technology, Pharmacy Collage, Damascus University Syria; Department of Chemistry and Materials, Imperial College London London SW7 2AZ UK; Department of Molecular Biology and Biotechnology, Atomic Energy Commission of Syria Damascus Syria hgkafa@gmail.com +963-940749813; Institute of Neurology, University College London Queen Square London WC1N 3BG UK

## Abstract

The use of nanoparticles (NPs) for intranasal (IN) drug delivery to the brain represents a hopeful strategy to enhance brain targeting of anti-epileptic drugs. In the present work, chitosan–lecithin NPs loaded with phenytoin (PHT), were prepared using the nano-precipitation method. The spherical nature of the NPs and their stability were confirmed using scanning and transmission electron microscopy, while the average dynamic size and zeta potential were measured using dynamic light scattering. The encapsulation efficiency of PHT was higher than 60% for all prepared NPs. Release studies showed that the amount of released PHT was directly related to the amount of chitosan used. The optimum preparation, L_10_C_i_^+^ was administered *via* the IN route, and the levels of PHT in the brain were measured in three-time points. Two experimental controls were given *via* the intraperitoneal (IP) and IN routes. The highest PHT amount reaching 1.01 ± 0.55% for L_10_C_i_^+^, which was associated with a sustained release of PHT. These preliminary findings show that the IN delivery of PHT-loaded NPs is very promising for managing epilepsy. The direct nose-to-brain approach increases the safety margins of PHT, while the sustained release could improve patient compliance in a clinical setting.

## Abstract 2

The use of nanoparticles (NPs) for intranasal (IN) drug delivery to the brain represents a hopeful strategy to enhance brain targeting of anti-epileptic drugs.

## Graphical abstract

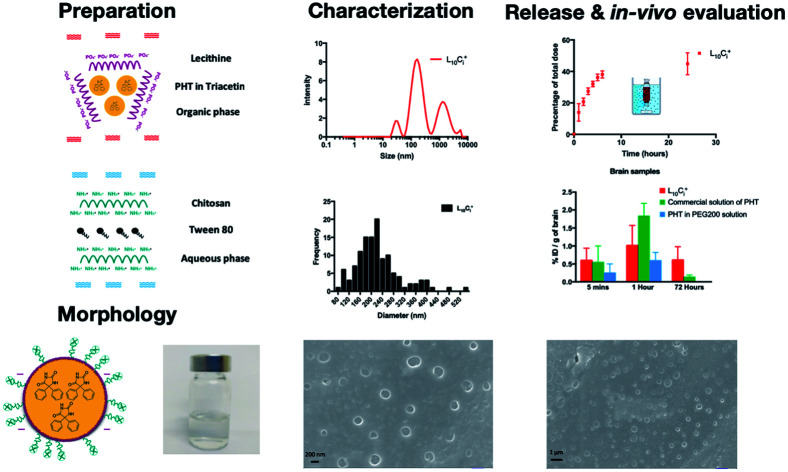

## 1. Introduction

Epilepsy is the third most common neurological disorder affecting about 65 million people around the world.^1^ Epilepsy is a chronic neurological disorder caused by excessive abnormal neuronal activity in the brain, which leads to seizures that influence the quality of life.^2^ Seizures may be focal or generalised depending on the location of the neural discharge and the spreading pattern.^3–5^ Both genetic and epigenetic mechanisms could be related to the alterations in voltage-gated and receptor-gated ion channels. For example, the up regulation of the T-type calcium channels increases the intrinsic burst firing of pyramidal neurons, which promotes seizures. Additionally, mutations in sodium channels are responsible for genetic epilepsy syndrome with a wide range of severities.^6,7^

Phenytoin (PHT) is the commonly used drug to manage epilepsy. PHT lowers the frequency and severity of partial and generalised tonic–clonic seizures activity in the motor cortex. PHT has an important role in the treatment of status epilepticus, and is also used for long term maintenance for epileptics. The main advantage of PHT is the ability to prevent status and grand mal seizures without causing drowsiness like the majority of other anti-epileptic drugs^8,9^ The mechanism of action relies on the reduction of the neural sodium channels efflux and blocking the T-type calcium channels.^6,10^ The main problem with PHT, however, is the extremely low aqueous solubility and the tendency to crystallize leading to precipitation in biological milieu. PHT precipitation in stomach medium causes slow and variable absorption. Additionally, the formation of needle-shaped crystals of PHT in the bloodstream post-injection causes pain, burning sensation and phlebitis. Moreover, PHT is highly protein bound leading to reduced bioavailability in the blood following *i.v.* administration. PHT is also a substrate to the P-glycoprotein (P-gp) efflux transporters in brain endothelial cells, which hinders the accumulation of this small and lipophilic drug into the brain and causes drug resistance in 30% of patients.^11^ Accordingly, PHT is administered in extremely high doses (300–400 mg kg^−1^ per day) to reach the therapeutic range in plasma.^12^ Such extremely high doses are associated with several serious side effects such as megaloblastic anaemia, decreased serum folate level, decreased bone mineral content, liver disease, cerebellar syndrome and gingival enlargement.^13,14^

Intranasal (IN) drug delivery is an emerging non-invasive method to deliver medications to the brain. The main advantage of employing the nasal route for drug delivery is the close association between the brain and nasal mucosa *via* the presence of the neural pathway including the olfactory and trigeminal nerve networks. Moreover, the nasal route avoids the first pass metabolism in the liver, and could be used for medications that are not suitable for more common delivery routes. However, the low surface area of nasal mucosa, the small volume that could be administrated and the rapid mucociliary clearance hinder the delivery of medications in their unaltered form, and requires the use of novel delivery systems to over come the nasal route limitations.^15^

Nanoparticles (NPs) have attracted significant attention owing to their unique physical and chemical properties that allow them to encapsulate, protect and control the targeted release of drugs in target tissues.^16^ Chitosan is a natural biodegradable polymer that is routinely used in the preparation of polymeric NPs for the delivery of neurological and anticancer drugs *via* the nasal route to the brain.^17^ The chitosan on the surface of NPs imparts important mucoadhesive properties,^18,19^ which reduce NPs clearance from nasal cavity and increase permeation across cellular membranes.^17,20^

In recent years, a number of promising drug delivery carriers have been developed to transport PHT to the brain. One such example included the use of electro-responsive hydrogel NPs (ERHNPs) modified with the brain-targeting angiopep-2 peptide. ERHNPs were able to lower the severity of the induced seizure at a high dose (20 mg kg^−1^) after intraperitoneal injection and subsequent drug release due to external electrical fields.^21^ Another attempt included the formulation of PHT-loaded microemulsion. The brain uptake following IN administration showed a rapid nose-to-brain transport of PHT compared with PHT solution in PEG 400 given IP. The amount of PHT in brain was 0.3 and 0.15 μg g^−1^ after 15 min of IN and IP administration, respectively.^22^ These studies showed the feasibility of employing IN administration for the delivery of PHT to the brain.

Chitosan–Lecithin NPs were successfully applied as a delivery system for many drugs using intranasal delivery route. Chitosan NPs enhanced the brain delivery and therefore therapeutic efficacy of anti-caspase peptide. The prepared chitosan NPs were rapidly detected in brain tissue compared with blank nanospheres functionalized with anti-mouse transferrin receptor monoclonal antibody (TfRMAb) and nanospheres lacking TfRMAb, suggesting that inhibition of caspase activity were due to efficient penetration of the peptide into brain using chitosan nanospheres.^23^ In another study, carboxymethyl chitosan NPs were used to encapsulate the anti-epileptic drug, carbamazepine, for nasal drug administration. The study confirmed a direct transport of carbamazepine to brain tissue from the nasal cavity compared with the conventional oral and intravenous drug formulations.^24^ Positively charged biocompatible polymers, such as chitosan, impart mucoadhesive properties that are vital for a successful IN delivery system. The mucoadhesive properties are important to avoid mucociliary clearance and increase residence time in the nasal cavity by the electrostatic interaction between the positively charged amine groups of d-glucosamine molecules of chitosan and the negatively charged sialic acid residues of mucin.^25^ On the other hand, the positive surface charge is a good indicator that the chitosan was present at the external interface of the NPs. As a result, smaller aggregates will be formed due to the repulsive positive forces between the NPs.^26^

To our knowledge, no study was carried out to prepare PHT-loaded chitosan lecithin NPs for intranasal delivery. Therefore, we prepared PHT-loaded NPs using the nano precipitation method. Lipid-core NPs were prepared by interfacial deposition of polymer using triacetin as the lipid dispersion in the core, lecithin as the polymer wall, tween 80 as stabiliser, chitosan was used as the coating material to obtain the positive charge for nanoparticles. The amount of chitosan, lecithin, PHT, the type of surfactant and the method of adding triacetin were optimised to achieve smallest size and highest drug loading in view of nasal-brain drug delivery. L_10_C_i_^+^ was selected as the most suitable candidate out of the tested NPs and was, therefore, used in all subsequent experiments. L_10_C_i_^+^ was investigated for its ability to delivery PHT to the brain in comparison to commercial solution of PHT and PHT solution in PEG200. Bio-distribution of PHT in brain and plasma was investigated from 5 minutes to 72 hours to evaluate the idea of using the prepared NPs for managing epilepsy.

## 2. Materials and methods

### 2.1. Materials

PHT (PHT), 5,5-diphenylhydantoin (batch #PB/10/14) was purchased from JPN PHARMA, India. PHT IV solution (50 mg ml^−1^) (batch #4RT007) was purchased from Pfizer, United States. Chitosan low molecular weight (batch #STBF8219V), acetic acid, triacetin (batch #MKBC5147), poloxamer 188, dialysis sacks (MWCO 12 000 Da) (batch #SLBQ 4638V) were purchased from Sigma-Aldrich Co., Germany. Lecithin (phosphatidylcholine 51.9% and phosphatidylethanolamine 12.5%) (batch #20617HHFEA) was supplied by Cargill Co., Germany. Acetone, ethanol were purchased from Eurolab, UK. Methanol, chloroform were purchased from Merck, Germany. Tween 80, tween 20 were purchased from AppliChem, Germany.

### 2.2. The preparation of chitosan–lecithin NPs

**Scheme 1:**
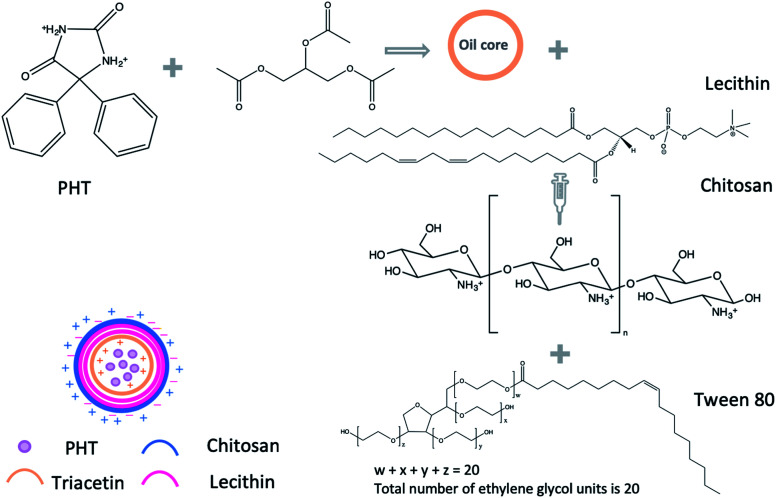
Synthesis of PHT loaded NPs.

Chitosan NPs were prepared using the method established by Sonvico *et al.*, with some modifications.^18^ Briefly, different amounts of lecithin (5, 10, 25 mg) were dissolved in 5 ml of ethanol : acetone solution (40 : 60% v/v). PHT (300, 600, 1200 μg) was dissolved in 200 μL triacetin, and added to the organic phase. Different amounts of chitosan (0.1, 0.25, 0.5, 1.25, 5, 20 mg) were suspended in 10 ml of deionised water. The solution was acidified using acetic acid (1 : 1.75 chitosan : acetic acid w/v). Unionised, hydrophilic surfactant (tween 20, tween 80 or poloxamer 188) was added to the aqueous solution of chitosan (0.2%) as described in Table 1. The organic phase containing lecithin and PHT was added drop-wise into the aqueous phase containing chitosan and stirred using magnetic stirrer (600 rpm in room temperature). The suspension was evaporated under low pressure using rotary evaporator (vacuum of 168 mbar, 70 °C, BÜTHI). To separate the precipitate residues, the NPs suspension was centrifuged using a refrigerated centrifuge (3214*g*, 15 min, 4 °C, Eppendorf, USA). The precipitate was dissolved in 1 ml of methanol and analysed using high-performance thing-layer chromatography (HP-TLC). The NPs suspension was placed in Viva-spin 100 kDa MWCO centrifugal concentrators (Sartorius, New York) to separate NPs from soluble non-reactive components. The NPs suspension was ultra-filtrated at 3214 g for 2 hours in 20 °C and NPs were collected from upper chamber of viva-spin tubes (Scheme 1).

**Table 1:** The composition of prepared PHT-loaded NPs

NP#	Chitosan (mg)	Lecithin (mg)	L/CS ratio	Phenytoin (mg)	Surfactant	Method of adding PHT & triacetin to organic phase
L_5_C_i_^+^	0.1	5	50	1.2	Tween 80	Phenytoin was added as a solute in triacetin
L_5_C_ii_^+^	0.25		20			
L_5_C_iii_^+^	20		4			
L_10_C_i_^+^	1.25	1.0	8			
L_10_C_ii_^+^	5		2			
L_10_C_iii_^+^	20		2.5			
L_25_C_i_^+^	0.5	25	50			
L_25_C_ii_^+^	1.25		20			
L_25_C_iii_^+^	20		1.25			
L_35_C_iii_^+^		35	1.25			
L_50_C_iii_^+^		50	1.25			
L_25_C_iii_T_20_^+^		25	1.25		Tween 20	
L_25_C_iii_PX_188_			1.25		Poloxamer 188	
L_25_C_iii_P_3_^+^			1.25	0.3	Tween 80	
L_25_C_iii_P_6_^+^			1.25	0.6		
LC_iii_O^−^			1.25	1.2		Triacetin was not added
L_25_C_iii_O_s_			1.25			Phenytoin and triacetin were added separately

### 2.3. The average dynamic size, PDI and zeta potential measurements

The average dynamic size (ADS) and the size distribution of NPs suspension were measured using dynamic light scattering (DLS) (Malvern Zetasizer, UK). NPs suspension was diluted with deionised water 50 times before measurement. The measurements were performed at 25 °C, collecting scattered light at an angle of 90° for 2 min, and the hydrodynamic size was presented as the average value of 20 runs, with triplicate measurements within each run. The size profiles were plotted against intensity of light scattered.

The surface charge (zeta potential) of NPs was also measured using DLS analysis. NPs suspension was diluted 50 times as before, and analysed in Zeta mode. The quantification was performed at 25 °C and the zeta potential was presented as the average value of 20 runs for each sample, with triplicate measurements within each run.

### 2.4. The encapsulation efficiency of PHT in NPs

The un-encapsulated PHT in the clear filtrate was analysed using HP-TLC, following the method described by Pippenger *et al.*, with some modification.^27^ 20 μL of samples were spotted mechanically 1.2 cm apart and 1 cm from the bottom of the silica gel-coated aluminium TLC plate (CAMAG automatic TLC sampler 4, Germany). The plate was then developed in chloroform : acetone 9 : 1 (v/v) and the UV absorption of tracks was measured at 217 nm using the HP-TLC scanner (CAMAG TLC scanner 3, Germany). The concentration of PHT was calculated from a linear standard curve. The EE% was calculated using the following equation:
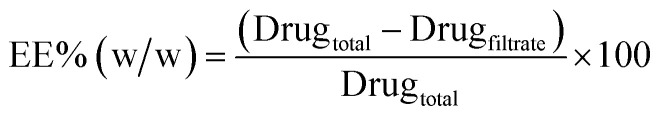


### 2.5. Release profile

NPs were suspended in 5 ml of deionised water, placed in dialysis sacks with a cut-off at 12 kDa (Sigma-Aldrich) and dialysed against 50 ml of deionised water. At each time point the release medium was collected and replenished. All samples were evaporated under low pressure using rotary evaporator (vacuum 72 mbar, water bath 80 °C) until complete dryness. The samples were re-hydrated using 2 ml of methanol, and analysed using HP-TLC. Data was showed as an accumulation curve.

### 2.6. Transmission electron microscopy (TEM) of NPs suspension

TEM was used to analyse the morphology of prepared NPs. A drop of sample suspension was placed onto a carbon/formvar-coated 300-mesh copper grids placed onto filter paper. The grids were then stained with uranyl acetate, and washed twice with 1 : 1 methanol : water solution to remove excess stain from the grids. The TEM bright field micrographs were obtained using 80 kV acceleration voltage and the appropriate objective aperture (Philips CM12).

### 2.7. Scanning electron microscopy (SEM) of NPs suspension

SEM imaging was employed to analyse the morphology of prepared NPs and to plot the size distribution histograms of NPs. Briefly, NP suspensions were diluted ten times with deionised water. One drop of sample was placed on the aluminium stub without any staining or coating. The samples were analysed using the (VIGA II Xmu, TESCAN, Chic) operating at an accelerating voltage of 20 kV. The images were obtained using a secondary electron detector with magnification ranging from to and 3000 kV and scan speed of 8.

### 2.8. The stability of the prepared NPs

The stability of the prepared NPs was studied on aqueous nano suspensions that were stored in 20 °C for three months. The morphology of the NPs was examined using TEM imaging immediately after preparation and then re-examined after the extended storage period. The stability of the prepared NPs in aqueous medium was studied in water with pH ranging from pH 3 to pH 9 at 20 °C for one week. The morphology of the NPs was examined using SEM imaging after the storage period.

### 2.9. Intranasal administration of NPs suspension

The non-invasive intranasal administration was carried out on conscious mice to take advantage of natural reflexes to inhale the dose internally as described in Hanson *et al.*, work.^20^ The advantage of this dosing method is to avoid complex invasive administration, which could lead to swelling and discomfort to the mice. The omission of the anaesthesia also minimises undue stress to the mice. Briefly, 15 μL of NPs suspension was dropped in each open nostril of conscious mouse enabling the delivery of the NPs suspension towards the roof of the nasal cavity. This drop-by-drop approach was repeated three times per mouse until the total volume of required dose (60 μL NPs suspension with PHT amount equivalent to 15 mg kg^−1^) is administered within 5 minutes. The division of the dose into multiple drops aimed to minimise mucociliary clearance, and avoid drainage into the nasopharynx.

### 2.10. The brain delivery of PHT using NPs suspension following intranasal administration

All animal procedures were performed in accordance with the Guidelines for Care and Use of Laboratory Animals of Breeding Unit for Inbred Mice at the Department of Molecular Biology and Biotechnology, Atomic Energy Commission of Syria, and approved by the Animal Ethics Committee of Damascus University.

In the first stage of this experiment, the optimal NPs suspension for brain delivery, *via* the nasal cavity, was investigated among five of the best preparations. As such, fifteen healthy female mice (BALB/c), aged 12–16 weeks, weighing 20–30 g were randomly divided into five groups. NPs (L_5_C_i_^+^, L_5_C_ii_^+^, L_10_C_i_^+^, L_25_C_ii_^+^, L_25_C_ii_^+^), with encapsulated dose of 7 mg PHT for every 1 ml of NPs suspension, were administered through the nasal cavity (60 μL NPs suspension with PHT amount equivalent to 15 mg kg^−1^) as detailed in Section 2.9.

Based on the evidence obtained in the characterisation experiments coupled with findings obtained from this *in vivo* experiment, L_10_C_i_^+^ was used in all subsequent experiments to investigate brain delivery of NPs. PHT brain delivery using L_10_C_i_^+^ was investigated in nine healthy female mice aged 12–16 weeks weighing 20–30 g randomly divided into three groups. L_10_C_i_^+^, with encapsulated dose of 7 mg PHT for every 1 ml of NPs suspension, was administered through the nasal cavity (60 μL NPs suspension with PHT amount equivalent to 15 mg kg^−1^) to the first group. Commercial solution of PHT (50 mg ml^−1^), used as a positive experimental control, was administered IP to the second group. PHT in PEG200 solution (7 mg ml^−1^), also used as an experimental control, was administered through the nasal cavity (60 μL, 15 mg kg^−1^) to the third group of mice. The negative control group consisted of six untreated mice.

### 2.11. The extraction of PHT from tissues and HPLC analysis

At the conclusion of each time point in the experiment, mice were euthanised using IP terminal anaesthesia containing 87.5 and 12.5 mg kg^−1^ ketamine and xylazine, respectively. Blood samples were obtained by open cardiac puncture, and the brain was then harvested and weighted. PHT was extracted from samples by liquid–liquid extraction (LLE) method. Briefly, blood samples (1 ml) were centrifuged at 2469*g* × 15 minutes (MPW-260R, Poland) to separate the serum layer. Serum was diluted with 10 ml methanol. The collected brain tissues were homogenised (25 000 rpm) in 10 ml methanol. Three groups of brain samples from untreated mice were spiked with PHT solution (25 μg ml^−1^) as a positive control. Samples were sonicated using a tip sonicator, 1 cycle and 100% amplitude (Sartorius AG, Germany), and centrifuged at 3214*g* × 15 minutes at 4 °C. The supernatants were collected, concentrated until total dryness (Eppendorf concentrator 5301, Germany) and resuspended in 200 μL methanol. The samples were centrifuged 18 188*g* × 30 minutes at 4 °C and the supernatants were filtered using Minisart® syringe Filter 0.45 μm prior to injection into the HPLC system. The concentration of PHT in biological tissues was measured using JASCO HPLC with UV/Vis detector, Japan. The concentration of PHT was measured using the following method described by Ningrum *et al.*,^28^ the analysis was conducted using methanol : water (55 : 45) as mobile phase, C8 (4.6 × 250 mm; 5 μm) as the stationary phase, 40 °C, 1 ml min^−1^ flow rate and the resolved peaks were measured at 210 nm wavelength.

### 2.12. Data analysis

The results are expressed as percentage injected dose per organ (% ID, mean ± SD, *n* = 3) or percentage injected dose per gram of tissue (% ID per g, mean ± SD, *n* = 3) and statistically analysed using one-way ANOVA.

### 2.13. Statistical analysis

All of experiments conducted in this work were repeated three times at least. To compare values obtained from the studied samples, analysis of variance (ANOVA) one-way as statistical model was performed using Prism v 7.0 a software (GraphPad Statistics, USA), *p < 0.05, **p < 0.01, ***p < 0.001.

## 3. Results

### 3.1. Linear regression and calibration curves

To approve the linearity of analysis methods (HP-TLC), the calibration curve height of absorbance peaks as a function of phenytoin concentration was plotted. The heights of symmetrical and well-resolved peak of phenytoin at Rf values of 0.2 against the concentration of phenytoin was linear in the range of (0–70) μg ml^−1^ or (0–140) ng per spot. Regression equation was “*Y* = 4.221 × *X* − 3.446” and correlation coefficient was 0.9966. Residuals at each point as the difference between the actual *y* value at a point and the estimated *y* value from the regression line were within 5% errors of the predicted values (95% confidence interval). Points look randomly scattered around zero. No evidence of nonlinear pattern or unequal variances.

**Fig. 1:**
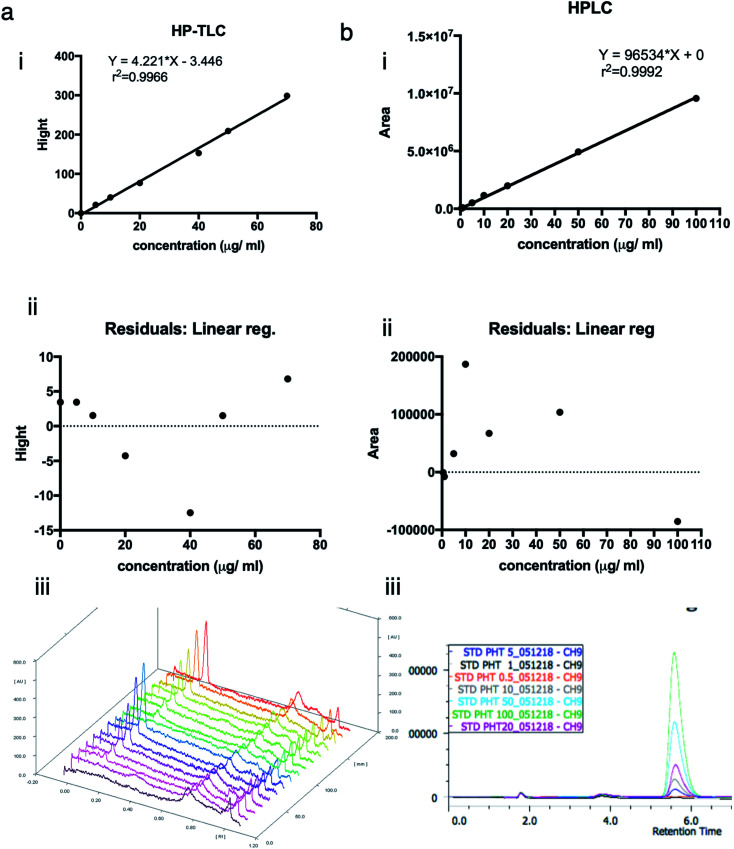
The calibration curves, residuals and chromographs of PHT concentration using HP-TLC and HPLC. (a.i) the calibration curve used for HP-TLC measurements relying on the height of the absorbance peaks against the concentration of PHT. The standard curve was linear in the range of 0–70 μg ml^−1^. (a.ii) Linear regression residuals were within 5% errors of the predicted values (95% confidence interval). (a.iii) The mobile phase for HP-TLC analysis was composed of chloroform : acetone (36 : 4%, v/v), which resulted in a sharp, symmetrical and well-resolved peak of PHT at Rf values of 0.2. (b.i) The calibration curve used for HPLC measurements relying on the area of the absorbance peaks against the concentration of PHT. The standard curve was linear in the range of 0–100 μg ml^−1^. (b.ii) The regression residuals showing no evidence of nonlinear pattern or unequal variances. (b.iii) The mobile phase for HPLC analysis was composed of methanol : water (55 : 45%, v/v), which resulted in a sharp, symmetrical and well-resolved peak of PHT at retention time of 5.6 minutes.

To approve the linearity of analysis methods (HPLC), the calibration curve area of absorbance peaks as a function of phenytoin concentration was plotted. The mobile phase composed of methanol : water (55 : 45%, v/v) resulted in a sharp, symmetrical and well-resolved peak of phenytoin at retention time of 5.6. The area of peaks against the concentration of phenytoin was linear in the range of (0–100) μg ml^−1^. Regression equation was “*Y* = 96 534 × *X* + 0” and correlation coefficient was 0.9992. Residuals at each point as the difference between the actual *y* value at a point and the estimated *y* value from the regression line were within 0.2% errors of the predicted values (95% confidence interval). Points look randomly scattered around zero. No evidence of nonlinear pattern or unequal variances (Fig. 1)

### 3.2. The physicochemical characterisation of PHT-loaded chitosan–lecithin NPs

#### 3.2.1. The visual appearance of the NPs suspension

The lack of solubility in aqueous media remains the biggest challenge for using PHT in biological systems. NPs were exploited to carry PHT as cargo in the oil core aiming to prevent crystallisation and to keep PHT available for treatment.

**Fig. 2:**
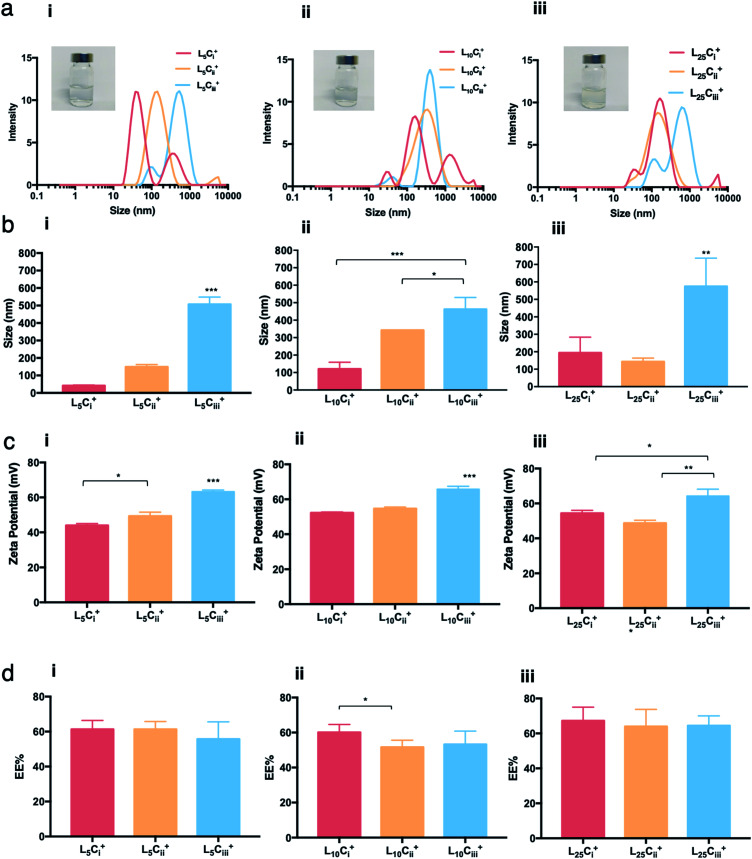
The characterisation of NPs with different amounts of chitosan using DLS and HP-TLC. The size distribution histograms (a) showing a dominant size population for each NP preparation, and the ADS (b) showed a significant increase in the size of prepared NPs as a function of the chitosan amount used in the preparation. (c) Zeta potential measurements showing that all prepared NPs carried a positive charge, which increased as the chitosan amount increased. (d) HP-TLC showed that the EE% of was independent of the chitosan amount in all NP preparations (one-way ANOVA test; *p < 0.05, **p < 0.01, ***p < 0.001, *n* = 3 at least).

**Fig. 3:**
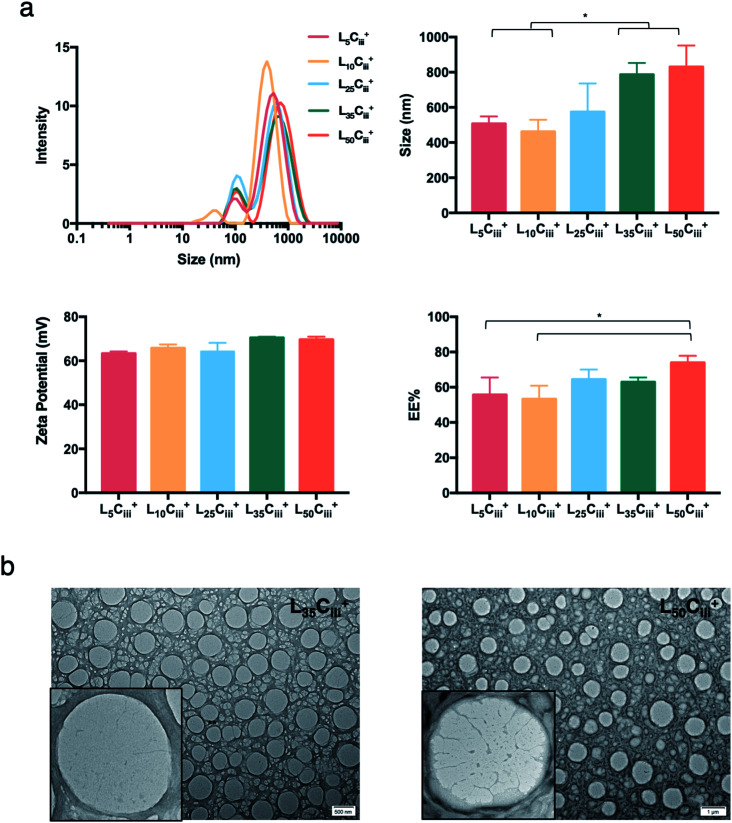
The effect of lecithin on the prepared NPs. (a) NPs size distribution histogram showing a dominant population with a narrow distribution for all NPs preparation regardless of the lecithin amount. ADS, zeta potential and EE% showed no significant changes until large amounts of lecithin (35 and 50 mg) were used in the preparation. (one-way ANOVA test; *p < 0.05, **p < 0.01, ***p < 0.001, *n* = 3 at least). (b) TEM micrographs of NPs prepared with 35 and 50 mg of lecithin.

**Fig. 4:**
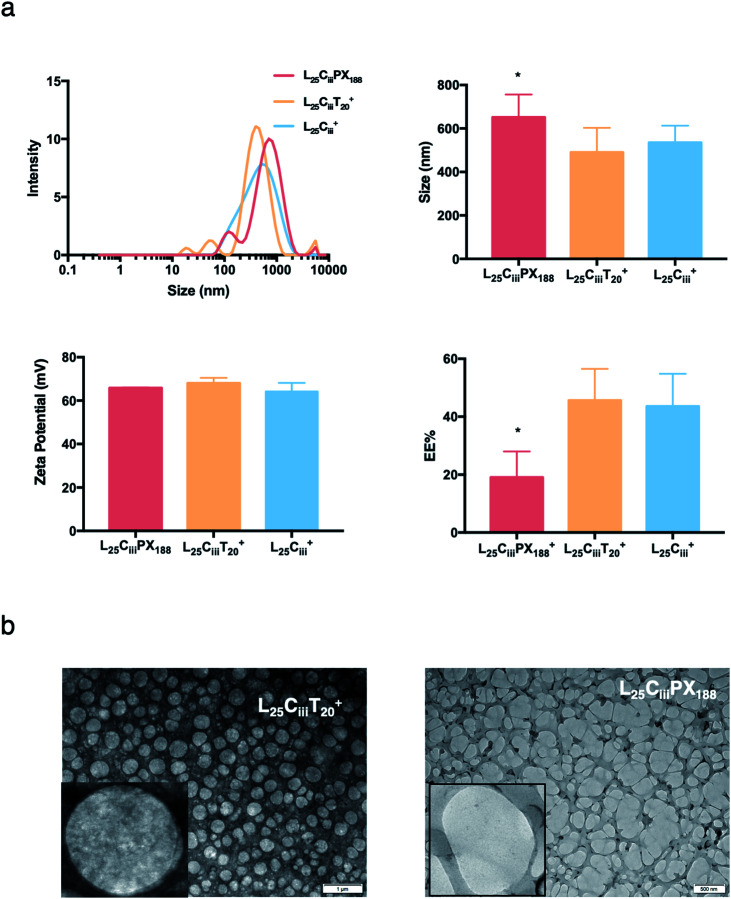
The effect of surfactants on the properties of prepared NPs. (a) NPs size distribution histograms showing highest homogeneity when tween 80 was used as a surfactant with one major population compared to multiple populations observed with tween 20 and poloxamer 188. ADS and zeta-potential measurements were comparable for all three tested surfactants. However, the EE% was significantly lower when poloxamer 188 was used in the preparation of NPs (one-way ANOVA test; *p < 0.05, **p < 0.01, ***p < 0.001, *n* = 3 at least). (b) TEM micrographs showing the spherical shape of NPs prepared with tween 20 and poloxamer 188.

**Fig. 5:**
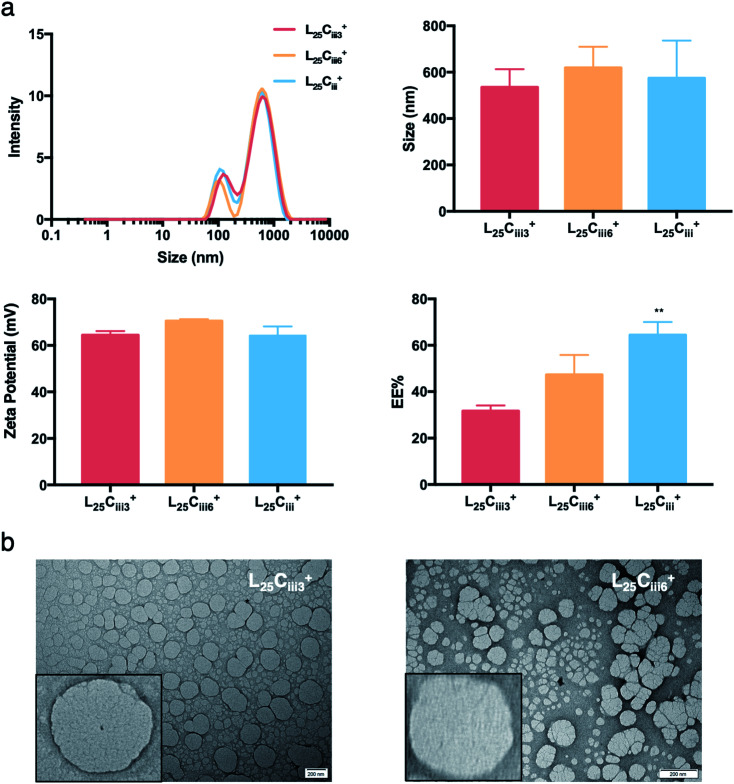
The effect of PHT amount of NPs properties. (a) NPs size distribution histograms showing narrow distribution for all prepared NPs regardless of the PHT amount. ADS and zeta-potential measurements were independent of the amount of PHT. The EE%, however, increased proportionally with the amount of PHT in the preparation reaching highest EE% with 1200 μg of PHT (one-way ANOVA test; *p < 0.05, **p < 0.01, ***p < 0.001, *n* = 3 at least). (b) TEM micrographs showing that NPs retained the spherical shape despite the increase in PHT amount.

The water dispersibility of the produced NPs was observed visually. Neither visual aggregations nor precipitations were observed in NPs suspensions when lecithin : chitosan ratio was below 50 and pH values of NP suspensions was between 4.5 and 5.0. Size distribution of NPs showed different populations with one major population per preparation. The reported size was calculated from the height of the main peaks (Fig. 2–5).

#### 3.2.2. The effect of studied factors on average dynamic size and PDI of NPs

The effect of various formulation parameters on the average dynamic size (ADS), surface charge and encapsulation efficiency of NPs were briefed in Table 2. ADS of prepared NPs was dependent on the amount of chitosan used in the preparation. As the chitosan amount increased the ADS of the NPs increased as well, but the size never exceeded 600 nm. The smallest ADS values were obtained with the least amount of chitosan (0.1 mg) reaching 41.8 nm for L_5_C_i_^+^. In contrast, all NPs prepared with high amount of chitosan (20 mg) showed a significant increase in size reaching 507, 462 and 572 nm for L_5_C_iii_^+^, L_10_C_iii_^+^ and L_25_C_iii_^+^, respectively (Fig. 2).

**Table 2:** Size, PDI, surface charge and EE% values of prepared PHT-loaded NPs (mean ± standard deviation; *n* = 6)

NP	*z* Average (nm)	PDI	Zeta potential (mV)	EE% ± SD	Average size
L_5_C_i_^+^	41.8 ± 3.46	0.343	43.96 ± 1.05	61.21 ± 5.12	—
L_5_C_ii_^+^	149.33 ± 12.7	0.297	49.33 ± 2.2	61.23 ± 4.44	—
L_5_C_iii_^+^	507 ± 41.56	0.457	63.26 ± 0.96	55.72 ± 9.8	343.3 ± 16.57
L_10_C_i_^+^	120.43 ± 38.43	0.495	52.23 ± 0.45	60.12 ± 4.4	222.36 ± 43.57
L_10_C_ii_^+^	342 ± 0.1	0.477	54.6 ± 0.96	51.61 ± 4.1	249.59 ± 26.9
L_10_C_iii_^+^	462 ± 67.54	0.47	65.7 ± 1.65	53.26 ± 7.5	300.4 ± 26.57
L_25_C_i_^+^	193.66 ± 90.23	0.43	54.36 ± 1.68	67.23 ± 7.8	370.57 ± 104.58
L_25_C_ii_^+^	142.66 ± 21.0	0.292	48.66 ± 1.68	63.98 ± 9.5	618.95 ± 281.95
L_25_C_iii_^+^	574.33 ± 161.87	0.537	64.23 ± 3.94	64.46 ± 5.6	1478.17 ± 20.83
L_35_C_iii_^+^	787.33 ± 65.24	0.601	70.53 ± 0.4	62.93 ± 2.6	—
L_50_C_iii_^+^	830.66 ± 121.59	0.608	69.63 ± 1.3	73.95 ± 3.91	772.73 ± 48.46
L_25_C_iii_T_20_^+^	490 ± 112.7	0.448	68 ± 2.47	45.62 ± 10.88	—
L_25_C_iii_PX_188_^+^	651.66 ± 104.5	0.483	65.83 ± 0.25	19.08 ± 8.87	—
L_25_C_iii3_^+^	535 ± 78.07	0.563	64.43 ± 1.72	31.71 ± 2.36	568.68 ± 28.39
L_25_C_iii6_^+^	619.33 ± 90.57	0.557	70.46 ± 0.8	47.28 ± 8.55	576.99 ± 334.71
L_25_C_iii_O^−^	281.66 ± 23.09	0.343	65.06 ± 1.85	26.59 ± 1.82	—
L_25_C_iii_O_s_	1030 ± 291.52	0.297	66.43 ± 1.75	31.39 ± 3.34	—

Increasing the lecithin amount from 5 mg in L_5_C_iii_^+^ to 25 mg in L_25_C_iii_^+^ did not influence ADS of NPs. However, there was a significant increase in ADS reaching 780 nm for L_35_C_iii_^+^ and L_50_C_iii_^+^, with 35 and 50 mg lecithin, respectively (Fig. 3).

The ADS of NPs prepared with tween 20 in L_25_C_iii_T_20_^+^ (490 nm) was lower than NPs prepared with tween 80 in L_25_C_iii_^+^ (574 nm). ADS increased when poloxamer 188 was used in L_25_C_iii_PX_188_^+^ reaching 651 nm as shown in Fig. 4.

The method of introducing PHT into the NPs core had a significant impact on the ADS of NPs. The addition of PHT into NPs core without using triacetin (L_25_C_iii_O^−^) resulted in an ADS of 281 nm. However, the size increased to 1030 nm in L_25_C_iii_O_s_ when PHT and triacetin were both present in the organic phase, but added separately. On the other hand, the ADS decreased to 574.33 nm when the PHT was dissolved in triacetin prior to the addition into the organic phase in L_25_C_iii_^+^ as shown in Fig. S7.†

The PDI values of the prepared NPs were less than 0.5 in all preparations regardless of the amounts of chitosan used. The lowest PDI value (0.290) was measured in L_5_C_ii_^+^ and L_25_C_ii_^+^ that have a CS : L ratio of 20. The one exception was found in L_25_C_iii_^+^ (PDI = 0.547) that has a CS : L ratio of 1.25.

In the respective preparations, as the lecithin amount increased the PDI increased. The PDI values were affected by the type of surfactant. The PDI was the lowest in NPs prepared with tween 20 (0.448) and increased to 0.483 and 0.587 in L_25_C_iii_PX_188_^+^ and L_25_C_iii_^+^, respectively.

ADS and PDI of NPs were independent of the amount of PHT added to the NPs preparation as detailed in Fig. 5.

#### 3.2.3. The effect of studied factors on surface charge of NPs

The chitosan chains imparted an overall positive charge to all prepared NPs. Fig. 2 shows that the higher the chitosan amount, the higher the zeta potential values regardless of the amount of lecithin used. The measured zeta potentials ranged from +43 to +64 mV. The increase in surface charge was only significant when the amount of chitosan reached 20 mg.

When the lecithin amount increased to 35, 50 mg in L_35_C_iii_^+^ and L_50_C_iii_^+^, respectively, the surface charge increased to +70 mV (Fig. 3). In all three tested surfactants, surface charge was ∼+65 mV, which indicated that the type of surfactant did not impact the surface charge of the prepared NPs (Fig. 4). Regardless of the method and the amount of PHT introduction into the NPs core, the positive surface charge of the prepared NPs remained +64 mV as detailed in Fig. 5 and S7.†

#### 3.2.4. The effect of studied factors on encapsulation efficiency (EE%) of NPs

The EE% of PHT within NPs ranged from 51 to 67% for all prepared NPs with different amount of chitosan. Increasing the chitosan amount from 0.1 to 20 mg did not lead to significant change in EE% (Fig. 2), which indicates that the EE% was independent of the amount of chitosan used. A significant decrease of EE% was only noticed when the chitosan amount increased from 1.25 mg in L_10_C_i_^+^ to 5 mg in L_10_C_ii_^+^.

Fig. 3 shows that increasing the lecithin amount from 5 to 35 mg did not impact the EE%, which ranged from ∼55 to 62% for L_5_C_iii_^+^ and L_35_C_iii_^+^. However, the EE% improved to 73.9% in L_50_C_iii_^+^. In terms of the used surfactant, L_25_C_iii_PX_188_^+^ showed significantly lower EE% (19%) compared with L_25_C_iii_T_20_^+^ (45%) or L_25_C_iii_^+^ (64%) as shown in Fig. 4.

On the other hand, the EE% of the NPs was dependent on the amount of PHT used in the preparation. As the PHT amount increased the EE% increased significantly as shown in Fig. 5. EE% was 31% in L_25_C_iii300_^+^, which improved to 64% in L_25_C_iii_^+^. Additionally, EE% of PHT was the highest when the PHT was dissolved in triacetin prior to the addition into the organic phase (Fig. S7†).

#### 3.2.5. Morphological analysis of NPs

**Fig. 6:**
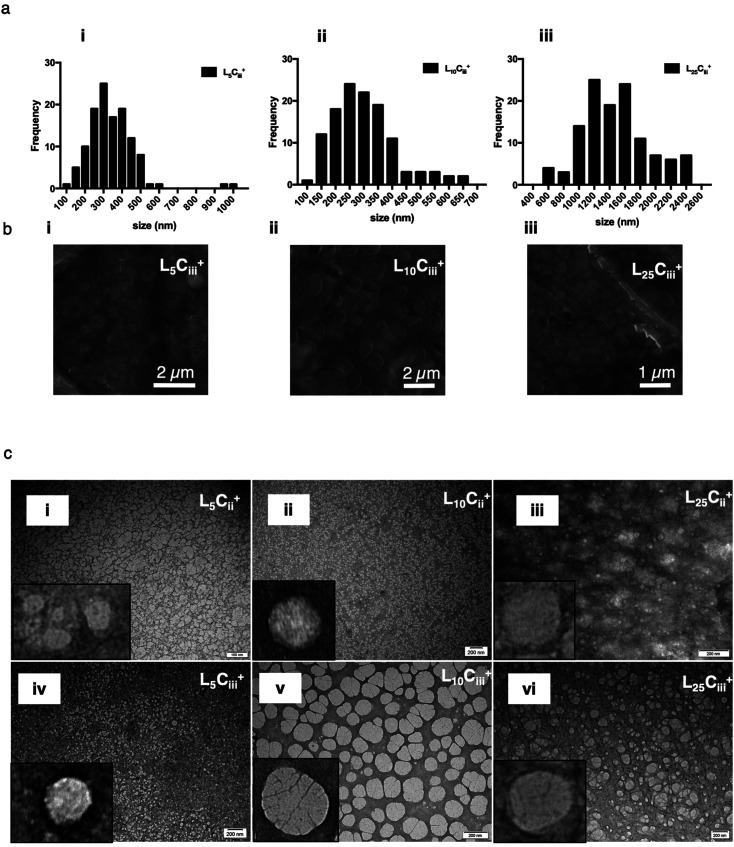
SEM size distribution histograms (a) for all prepared NPs generated from manually measuring the size of NPs from SEM micrographs (*n* = 120 at least). The spherical shape of the NPs were confirmed using uncoated SEM images (b), and bright field TEM images (c) on carbon/formvar-coated 300-mesh grids. The increase in chitosan amount in the NP preparations was associated with more defined NPs structure on the edges of the NPs.

All prepared NPs were characterized using SEM and TEM. The histograms (Fig. 6, S1†) reflected the narrow size distribution of NPs under vacuum. The average size calculated from SEM micrographs increased with increasing the chitosan amount, which agrees with hydrodynamic size measurements (Table 2). The only exception was related to NPs prepared with 25 mg of lecithin in L_25_C_ii_^+^ and L_25_C_iii_^+^ when a noticeable difference between hydrodynamic size and size under vacuum was observed. This difference in size could be attributed to the small population measured in the SEM histograms compared to DLS measurements, and the reliance on measuring large tangible NPs with more defined shapes when using the SEM. On the other hand, the histograms (Fig. S6†) reflected the wide size distribution of L_25_C_iii300_^+^ and L_25_C_iii600_^+^ under vacuum.

The TEM and SEM micrographs in Fig. 6 proved the uniform spherical shape of all prepared NPs with no deformity of its structure. The TEM micrographs revealed that as the chitosan and lecithin amounts increased, the morphology of NPs was more defined and the more detailed surface of NPs was observed as shown in Fig. 3, 6 and S2–S4.† TEM micrographs revealed the oval shape of L_25_C_iii_PX_188_^+^ prepared with poloxamer 188 as a surfactant (Fig. 4) and (Fig. S5†). The SEM and TEM micrographs (Fig. S7†) confirmed the formation of NPs without adding triacetin.

### 3.3. The release profile of PHT from NPs

**Fig. 7:**
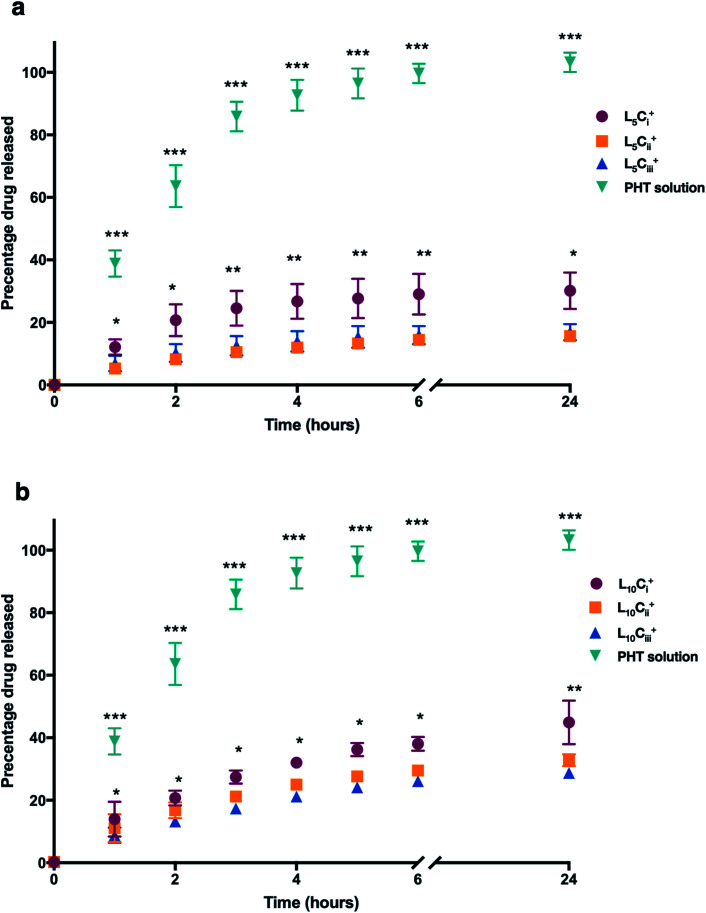
The release profile of PHT from NPs, which were dialysed against de-ionized water. A solution of PHT in water was used for comparison. The cumulative release curve of NPs containing 5 mg of lecithin (a) and 10 mg of lecithin (b) showed that the amount of PHT released was significantly higher in NPs with low amount of chitosan compared to other NPs at all tested time points. The PHT solution showed a rapid release of PHT (99%) within 6 hours. The results are represented as mean% ± SD (*n* = 3 at least) (one-way ANOVA test; *p < 0.05, **p < 0.01, ***p < 0.001).

The release profile for the prepared NPs was studied using different amounts of chitosan and lecithin. PHT water solution was used as a control for the experiment. The mean cumulative percentage of PHT released from NPs *versus* time plot is shown in Fig. 7. The amounts of PHT released reached 30%, 15% and 16% for L_5_C_i_^+^, L_5_C_ii_^+^ and L_5_C_iii_^+^ after 24 hours, respectively. In contrast, increasing the lecithin amount from 5 to 10 mg resulted in an increased release percentage reaching 44%, 32% and 28% for L_10_C_i_^+^, L_10_C_ii_^+^ and L_10_C_iii_^+^ after 24 hours, respectively.

The PHT release was significantly higher for NPs that contained the lowest amount of chitosan (L_5_C_i_^+^ and L_10_C_i_^+^) at all time points. PHT solution showed a rapid release of PHT (99%) within 6 hours. The rate of PHT release from PHT solution was consistent with a release profile of dissolved drugs while encapsulated PHT showed a slower release kinetics consistent with the rate of release from NPs, which was highly dependent on the amount of chitosan used.

### 3.4. The aqueous stability of NPs

L_25_C_iii_^+^, L_25_C_iii_T_20_^+^ and L_25_C_iii_PX_188_^+^ were stored as nano suspension in deionised water in room temperature for 3 months. The TEM micrographs of NPs post-storage confirmed that NPs retained their spherical structure despite the long storage period with no signs of deformation. The NPs appeared well dispersed with no apparent aggregation. Also, no polymeric precipitation was observed in the background despite careful examination. The dispersibility of the L_10_C_i_^+^ was observed visually. Slight precipitation was seen in pH 3 and dense aggregation and precipitation were observed in pH 7 and 9. However, neither aggregations nor precipitations were observed in pH 5, which was used in all further experiment (Fig. S8†)

### 3.5. The accumulation of PHT in mouse brain after IN administration

**Fig. 8:**
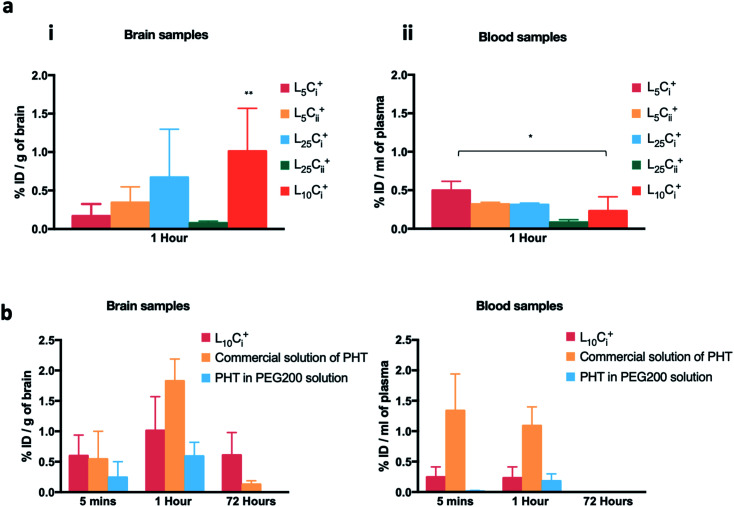
The accumulation of PHT in brain and blood following in administration. (a) The brain and blood accumulation of PHT following in administration of different NPs formulations showing that the highest PHT level in brain (1.01 ± 0.558% ID per g) was associated with L_10_C_i_^+^. (b) PHT biodistribution in brain and blood at 5 minutes, 1 and 72 h after the in administration of L_10_C_i_^+^ and the PEG200 solution, and the IP administration of commercial solution of PHT. The biodistribution profile in brain suggested a rapid accumulation of PHT in mouse brain after 5 minutes of dosing reaching maximum concentration after 1 hour for both L_10_C_i_^+^ and experimental controls. Significant amount of PHT was detected after 72 h with L_10_C_i_^+^ (0.607 ± 0.373% ID per g), while PHT amount decreased sharply after IP administration of PHT solution and PHT in PEG200. The results are represented as % ID per g, mean ± SD, (*n* = 3 at least) (*p < 0.05, **p < 0.01, ***p < 0.001).

The initial pilot experiment was designed to assay the most suitable NP preparation for the IN delivery of PHT to the brain, which would be used in all subsequent experiments. Fig. 8a shows that the highest PHT level in brain was associated L_10_C_i_^+^ after 1 hour of IN administration (1.01 ± 0.558% ID per g), and the lowest (0.077 ± 0.025% ID per g) was associated with L_25_C_ii_^+^. The level of PHT in the brain following the administration of other tested NPs were in the following order after 1 hour: L_25_C_i_^+^ > L_5_C_ii_^+^ > L_5_C_i_^+^. On the other hand, Fig. 8a shows that 0.23 ± 0.183% ID per g of PHT was circulating in the plasma after 1 hour of L_10_C_i_^+^ administration, which was close to the PHT amount in plasma after the IN administration of L_5_C_ii_ and L_25_C_i_^+^. Lower amount of PHT was detected in plasma after L_25_C_ii_^+^ administration (0.082 ± 0.036% ID per g) compared to that of L_10_C_i_^+^, while L_5_C_i_^+^ was associated with higher amount of circulating PHT compared to L_10_C_i_^+^.

Commercial solution of PHT, and PHT in PEG200 were used as positive controls in all *in vivo* experiment. The PHT bio-distribution profile in brain suggested a rapid accumulation of PHT in mouse brain at 5 minutes following the IN administration of NPs (0.59 ± 0.34% ID per g), the IP administration of commercial PHT solution (0.54 ± 0.45% ID per g) and IN administration of PHT in PEG200 solution (0.24 ± 0.2% ID per g).

The amount of PHT in the brain increased over time in all compared preparations. The highest brain accumulation was detected with IP administration (1.825 ± 0.363% ID per g) after 1 hour. The IN delivery of L_10_C_i_^+^ achieved comparable PHT accumulation in brain (1.0107 ± 0.55% ID per g) to IP administration. The lowest PHT amount was detected after the IN administration of PHT in PEG200 solution (0.591 ± 0.229% ID per g).

A significant amount of PHT was still detected after 72 hours with L_10_C_i_^+^ (0.607 ± 0.373% ID per g) suggesting a sustained concentration-time profiles in the brain. In contrast, the level of PHT in the brain decreased sharply (0.128 ± 0.059 ID per g) with IP administration after 72 hours, while no PHT was detected after 72 hours of PHT in PEG200 administration.

PHT amounts were measured in plasma after 5 minutes of IN dosing of NPs, PHT PEG200 solution and IP administration of PHT commercial solution as detailed in Fig. 8b.

The IN administration of NPs showed a significant reduction in PHT concentration in blood compared to IP administration at the early time points (0.2 ± 0.16 *vs.* 1.3 ± 0.6% ID per g of blood at 5 min, 0.23 ± 0.18 *vs.* 1.08 ± 0.3% ID per g of blood at 1 hour). However, almost no PHT was detected in plasma even after 1 hour of IN administration of PHT-PEG200 (0.179 ± 0.12% ID per g). The blood circulation profiles decreased over time until 72 hours after both IN and IP dosing.

## 4. Discussion

The treatment of seizure emergencies requires rapid delivery of anti-epileptic drugs to the brain in high doses. In order to reach this therapeutic range, PHT is administered *i.v.* in high doses, which causes serious side effects.^13,14,29^ The intranasal route could potentially be used as an alternative route for delivering anti-epileptic medications due to its non-invasive nature, potential for direct nose-to-brain delivery and avoidance of first-pass metabolism. However, the limited volume of the nasal cavity and poor water solubility of anti-epileptic drugs restrict absorption, leading to insufficient levels in the brain.^14,30^

In this work, PHT-loaded chitosan–lecithin NPs were investigated for their ability to deliver PHT to the brain following IN administration. Chitosan–lecithin NPs were first prepared by Sonvico *et al.*, using the nano-precipitation of polymers dissolved in different mediums, where chitosan and lecithin are dissolved in acidic and organic medium, respectively.^18,31^ Gerelli *et al.*, investigated the interaction of chitosan and lecithin when forming NPs using cryo-TEM, which indicated the presence of a multi-lamellar structure of the lipid shell surrounded by positively charged chitosan molecules.^32^ The NPs structure achieved by electrostatic interaction between the positive chitosan and the negative lecithin.^18^

Low molecular weight chitosan was used in this study, which has been reported to have a higher aqueous solubility, and short polymer chains that contributes to forming smaller particles compared to medium and high molecular weight chitosan.^25,33,34^ The pH values of prepared NP suspensions were between 4.5 and 5.0, which is compatible with the biological pH of nasal mucous, and in agreement with previous study.^32^

In the present study, the ADS and zeta potentials of the prepared NPs were highly dependent on the amount of chitosan used. Lecithin caused an increase in ADS only when high amounts were used. These findings were in agreement with previously reported study where the size and the zeta-potential values of chitosan–lecithin NPs increased as a function of chitosan amount.^26^ Sonvico *et al.*, also showed that the size and surface charge of the NPs were dependent on the amount of chitosan in the aqueous solution, and the ratio of lecithin to chitosan in the preparation. The aggregation was evident in preparations having 30 : 1 and 40 : 1 lecithin : chitosan ratios, probably due to the neutralisation of the surface particle charge.^18^ Dammak *et al.*, has showed that the size and stability of colloidal NPs were determined by the net charge on the particle surface. When the zeta potential was largely positive, the size of particles was sub-micron and the colloidal suspension was stable due to the electric repulsion, which prevent aggregation and sticking NPs by keeping colloidal particles well separated from each other and from surfaces.^35^ As a result, the relatively high positive surface charge detected on the surface of NPs gave an explanation of high stability of prepared nano suspension.

Chitosan–lecithin NPs are biocompatible, biodegradable, and safe delivery system for poorly soluble drugs. Chitosan has been safely used as a penetration enhancer in conjugation with lecithin in several studies.^36–38^ Chitosan–lecithin NPs with surface charge approx +50 mV did not show cytotoxicity on MCF-7 cells, and the cells viability was not affected.^36^ Also Clementino *et al.*, evaluated the toxicity of chitosan–lecithin NPs with surface charge reaching +57 mV using RPMI 2651 nasal cell lines, and no signs of toxicity were reported up to 72 hours of exposure in static conditions.^37^ These reported findings supported the idea that chitosan–lecithin NPs are highly biocompatible, safe and suitable for nasal administration.^37,38^

IlK *et al.*, have previously showed that increasing the amount of kaempferol led to an increase in the size of chitosan–lecithin NPs.^39^ However, the data presented here showed the opposite effect as there was no significant increase in the size of NPs when higher amounts of PHT were used.

The EE% is generally dependent on the nature of the encapsulated drug mainly in terms of amount and charge^.^^40^ For example, Barbieri *et al.*, showed that the cationic drug tamoxifen competes with chitosan for the electrostatic interaction with phospholipids, and therefore the EE% was inversely proportional to the amount of tamoxifen in the preparation.^41^ Further evidence comes from the use of the neutral drug progesterone, which had no significant effect on the EE% in the preparation.^18^ However, in the present work, it was observed that the EE% increased when higher amounts of PHT was used in the preparation regardless of its neutral charge.

PDI values are used to determine the homogeneity of the prepared NPs.^39^ In this work, PDI values did not exceed 0.5 indicating an acceptable homogeneity for the prepared NPs. The PDI value only exceeded this threshold in one NPs preparation when the chitosan and lecithin amounts were 20 mg and 25 mg, respectively.

In our experiments, pharmaceutical lipophilic solvent (triacetin) was used as a vehicle to carry the PHT into the NPs core. The data showed that pre-mixing the PHT with triacetin resulted in an increase in EE%, this increase could be attributed to the fact that PHT is 200 times more soluble in triacetin than in water, and therefore triacetin could act as vehicle for ferrying PHT into the NPs core.^42^ Clementino *et al.*, has worked on Maisine® plus Labrafac™ as oil-based vehicle, which were used to formulate an oil core inside the prepared NPs. The use of oil core improved the EE% of simvastatin significantly, which agreed with our findings.^37^

The data presented here also shows that the EE% was independent of the chitosan amount in the NPs, which was in agreement with previously published work that attributed this observation to the presence of chitosan on the surface of the NPs.^18,39^ The used amount of lecithin did not affect the EE% when the lecithin amount did not exceed 20 mg. However, high amounts of lecithin resulted in an increase in EE%. Chevalier *et al.*, found that high lecithin amount produced a thick and strong layer adsorbed on the oily droplet surface, which could be the cause of this increase in EE%.^43^

Electron microscopy examination provided a solid evidence for the morphology of the prepared NPs. The NPs were homogeneous and spherical in shape with a compact core surrounded by a relatively thin outer shell attributable to a single bilayer structure. The NPs were also separate from each other in all our observations, and almost no aggregation was observed between the prepared NPs. The stability of the NPs in suspension and their uniform spherical shape could be a function of their positive surface charge as previously demonstrated.^39^ At pH values higher than 5, the amine groups on the chitosan backbone are deprotonated, which reduces the electrical repulsions between NPs. The reduction in electrical density could lead to the observed NPs aggregation, clustering and insoluble hydrogel formation between groups of NPs.^44^ On the other hand, an expected precipitation of PHT occur at pH 3 due the insoluble nature of PHT. NPs showed good stability over two storage times, 7 and 90 days, at pH 5 which is the optimum pH that matches the natural environment in the nasal cavity.^45^

The dialysis method was adapted in this work to monitor the release profile of PHT from the prepared NPs. The dialysis technique is considered the most suitable method for determining the drug release profile from drug delivery systems.^46^ One of the limitation of the dialysis method is the resistance of the dialysis membrane to the free movement of the drug through the pores.^46^ Therefore, the aqueous solution of PHT was used as a positive control in all release studies presented here. Deionised water (pH 5) was used as a release medium for two reasons. Firstly, the acidity of the release medium was selected to match that of nasal secretion.^47^ Secondly, the release medium was carefully selected and completely replenished at each time point to avoid the supersaturated state that is associated with PHT.^48^

PHT release profile from prepared NPs showed a biphasic release pattern: one initial burst release followed by a prolonged release. Similar to our findings, Clementino *et al.*,^49^ found the release rate of simvastatin from chitosan–lecithin NPs was also associated with an initial burst release attributed to rapid desorption and diffusion of simvastatin molecules located close to the surface of the NPs, followed by a slow release of the drug in the NPs core.

The observed release profile was a function of the two diffusional barriers within the PHT-loaded lipid core NPs. These diffusion barriers comprise the chitosan polymer wall and the lecithin layer surrounding the NPs oil core. Previous studies have demonstrated that the chitosan shell has an important role in controlling drug release. Moreover, NPs size also plays an important role in contouring drug release from NPs.^25^

The release studies concluded after 24 hours before reaching total PHT release. This behaviour was previously reported with encapsulated tamoxifen, which showed an extended release rate from chitosan–lecithin NPs even after 120 hours.^41^

The mechanistic release studies showed extensive evidence for the ability of chitosan layer to modify the release profile of PHT. Delated release was observed compared with PHT solution in all tested NPs. PHT release rate decreased with the increase of chitosan amount in NPs which agrees with Barbieri *et al.*,^41^ and Pergo *et al.*^50^

The ability of NPs to deliver PHT to the brain was tested *in vivo* using BALB/c mice. The experiments were designed to assess the amount of PHT that could reach the brain at different time points, and to investigate the best NPs with the highest PHT delivery to the brain. L_10_C_i_^+^ gave the highest PHT brain level compared with the other tested preparations and was selected as a candidate for all subsequent experiment.

Although PHT is available in oral dosage forms, IP administration of commercial solution achieves direct delivery to the systemic circulation.^51^ This reason make IP administration of PHT commercial solution an appropriate control to allow a direct comparison between systemic IP administration and the IN delivery of NPs. Moreover, to evaluate the polymeric NPs role in improving IN delivery and nasal residence time, PHT in PEG 200 solution was chosen as a control, which was also administrated *via* IN route.

Drug delivery to the brain through the IN route is known to vary depending on the nature of drugs used and the NP preparations. In addition to reaching the brain *via* accessing the systemic circulation and facing the BBB, some drugs are able to reach the brain directly through the olfactory neurons in the olfactory epithelium and/or the trigerminal nerve network.^52,53^ Carbamazepine, for example, was shown to enter the brain directly from the blood stream following IN administration into mouse. This was attributed to the quick absorption of carbamazepine to the blood stream through the nasal membrane.^51^ In the present work, however, the level of PHT in the brain was much higher compared with plasma level across all tested time points following IN administration of L_10_C_i_^+^, which could suggest that PHT was able to enter the brain directly through the nasal mucosa.

The direct brain entry of PHT after IN administration could be attributed to the surfactant tween 80, which was shown to improve the penetration of drug through nasal mucosa.^54^ Additionally, the positive charge and mucoadhesive properties of chitosan, could also facilitate the direct entry to the brain through the nasal mucosa as previously demonstrated.^25,55^ For example, carboxymethyl chitosan NPs were used as a carrier to deliver carbamazepine *via* the IN route. The data showed statistically higher amounts of carbamazepine in the brain when it was delivered encapsulated within carboxymethyl chitosan NPs *versus* that which was administered as a solution.^24^ The presence of high carbamazepine concentration in the brain compared with the plasma after IN administration was in agreement with our findings. As such, the use of chitosan as a mucoadhesive polymer in NPs was essential to achieve this direct delivery to the brain, which was probably dependent on the increase in residence time within the nasal cavity and avoiding the systemic circulation and/or pharynx drainage.^24,54^

Additionally, the level of PHT was high in the plasma in the early time points after IP administration. This is consistent with the pharmacokinetic profile of PHT following systemic administration and its ability to cross the BBB as a small lipophilic molecule (log *P* 2.47).^56^ In comparison, the IN administration of L_10_C_i_^+^ was associated with extremely low amount of PHT in the plasma and relatively high amount in brain, which provided further evidence that PHT reached the brain directly through the nasal cavity without reaching the systemic circulation.

One of the advantages of using NPs for the delivering PHT to the brain, was their ability to achieve sustained drug release even in extended time points. The amount of PHT that was delivered *via* the IP route decreased significantly in the brain after 72 hours. In contrast, substantial amounts of PHT were still present in the brain after 72 hours of IN administration of L_10_C_i_^+^. A similar observation was reported with lamotrigine-loaded lipid carriers in Wistar rat model that resulted in a sustained drug concentration in the brain 24 hours after IN administration. Scintigraphy studies proved the high accumulation of lamotrigine in brain after IN administration of NPs compared to IN and/or oral administration of lamotrigine solution.^54^ The sustained accumulation of PHT in the brain is very important to managing epilepsy and prevent seizures and the use of IN delivery in this case proved superior to the IP delivery.^57^

## 5. Conclusion

In this work, PHT-loaded NPs were prepared for managing epilepsy. The choice of IN delivery was largely due to the unique connection between the nasal cavity and the olfactory region in the brain, and the direct nose-to-brain drug delivery, which is important for anti-epileptic drugs that are associated with server side effects when administered systemically. The use of biocompatible polymers such as chitosan imparts mucoadhesive properties that are vital for a successful IN delivery system. The amount of chitosan was the main factor affecting both the ADS and zeta potential of NPs. The high EE% of PHT in all tested preparations was mainly due to the solubility of PHT in triacetin and using tween 80 as a surfactant. Imaging by TEM and SEM was employed to gain unequivocal evidence for the spherical shape of NPs, and release studies showed the ability of the prepared NPs to release PHT over time (44% of PHT was released from L_10_C_i_^+^ after 24 hours). We have also studied the accumulation of PHT in brain and plasma of BALBc mice after IN administration of five different NP preparations with suitable properties. L_10_C_i_^+^ was the optimum NPs preparation for further comparison with the commercial PHT and the PHT-PEG 200 solutions that were administered IP and IN, respectively. A rapid accumulation of PHT in mouse brain following the administration of both NPs and PHT solution was detected after 5 min and 1 hour. Sustained concentration-time profiles in the brain were observed for IN administration compared with IP administration at the late time point. Future work will focus on pharmacokinetic and bio-distribution of L_10_C_i_^+^ in major organs, and verifying the mechanism of direct brain entry after IN administration, which should paves the way for using NPs for the delivery of PHT to epilepsy sufferers with reduced side effects and increased compliance.

## Conflicts of interest

There are no conflicts to declare.

## Supplementary Material

RA-010-D0RA04890A-s001
