# Supplementary material for: Preparation and characterisation of PHT-loaded chitosan lecithin nanoparticles for intranasal drug delivery to the brain
Source: RSC Adv. 2020 Aug 5;10(48):28992–9009. doi: 10.1039/d0ra04890a (PMC9055806; doi:10.1039/d0ra04890a)
Supplement: RA-010-D0RA04890A-s001 [file RA-010-D0RA04890A-s001.pdf]

Electronic Supplementary Material (ESI) for RSC Advances.
This journal is © The Royal Society of Chemistry 2020

Preparation and Characterisation of PHT-Loaded Chitosan Lecithin Nanoparticles for Intranasal Drug Delivery to The Brain

Amal Yousfan[a], Noelia Rubio[b], Abdul Hakim Natouf[a], Aamal Daher[c], Nedal Al -Kafry[c], Kerrie Venner[d], Houmam Kafa[c]*

[a] Department of Pharmaceutics and Pharmaceutical Technology, Pharmacy Collage, Damascus University, Syria.

[b] Department of Chemistry and Materials Imperial College London London, SW7 2AZ, UK

[c] Department of Molecular Biology and Biotechnology, Atomic Energy Commission of Syria, Damascus, Syria.

[d] Institute of Neurology, University College London, Queen Square, London WC1N 3BG, UK.

* Corresponding author. Tel: +963940749813

E-mail address: hgkafa@gmail.com (Houmam Kafa)

Supplementary information

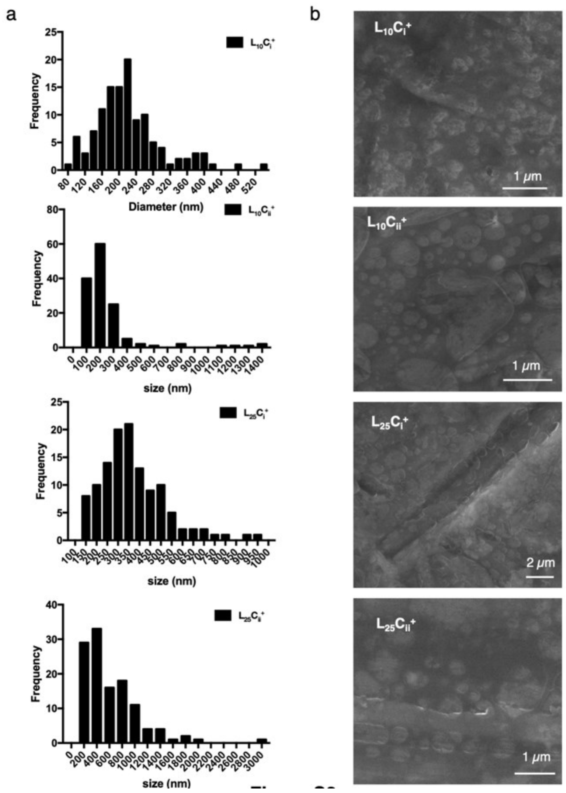

Figure S1. SEM characterization of PHT-loaded NPs. (a) SEM micrograph of NPs prepared with 10 and 25 mg of lecithin. **(b)** The size distribution histograms plotted after measuring the size of at least 120 NPs per group from SEM micrographs.

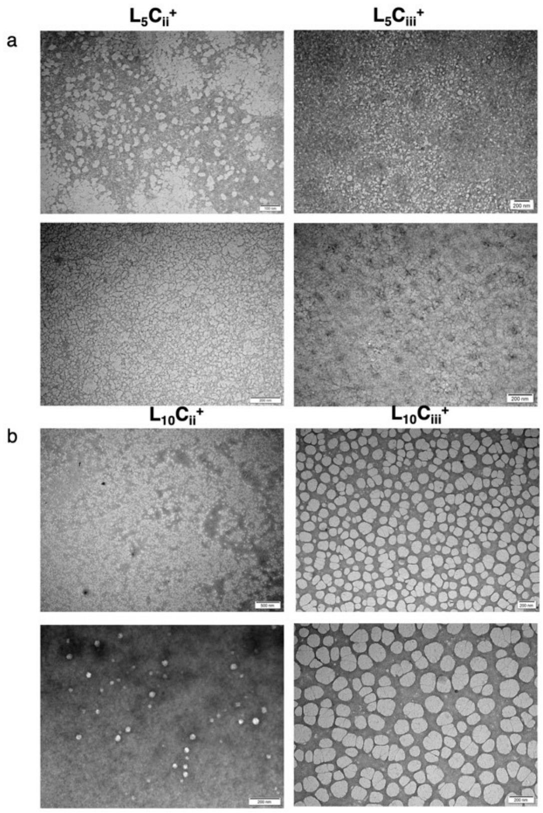

Figure S2. TEM micrographs of PHT-loaded NPs prepared with 5 and 10 mg of lecithin.

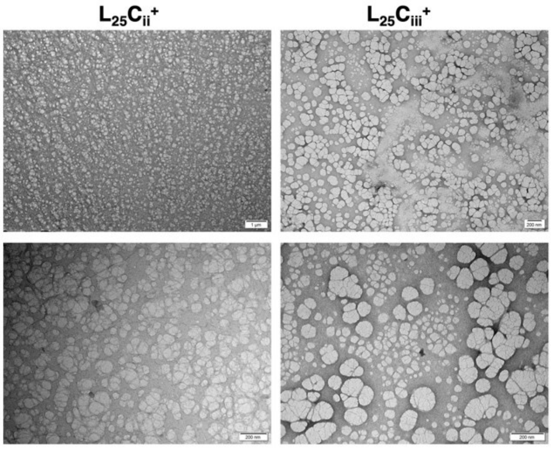

Figure S3. TEM micrographs of NPs prepared with 25 mg of lecithin.

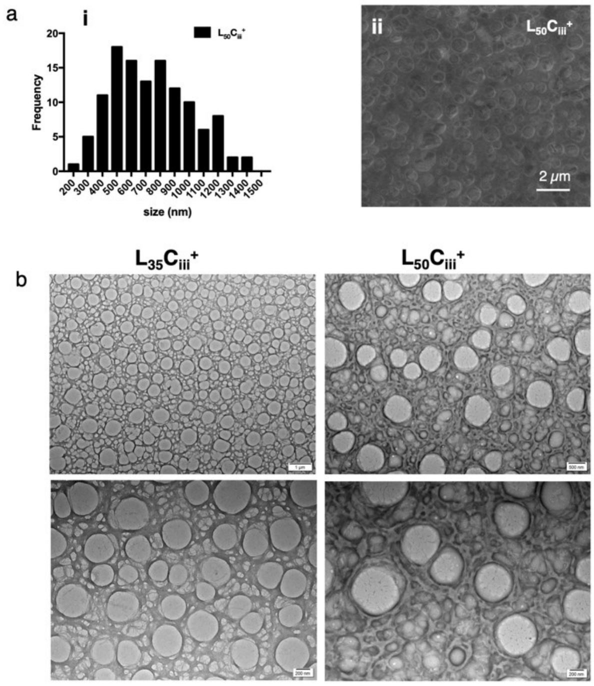

Figure S4. The effect of high amounts of lecithin on NPs. (a.i) The size distribution histograms plotted after measuring the size of at least 120 NPs per group from SEM micrographs. **(a. ii)** SEM micrographs of NPs prepared with 50 mg of lecithin. **(b)** TEM micrographs of NPs prepared with 35 and 50 mg of lecithin.

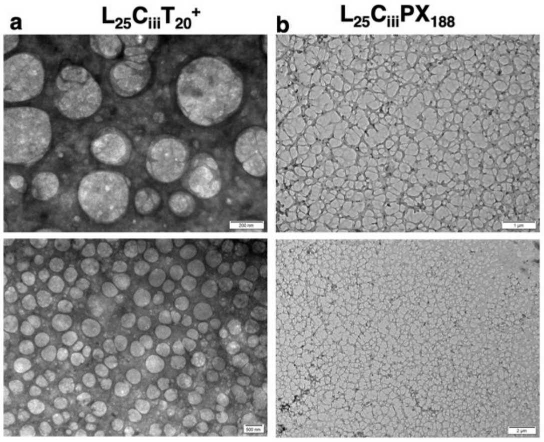

Figure S5. The effect of surfactant on NPs. TEM micrographs of NPs prepared with **(a)** Tween 20 and **(b)** Poloxamer 188.

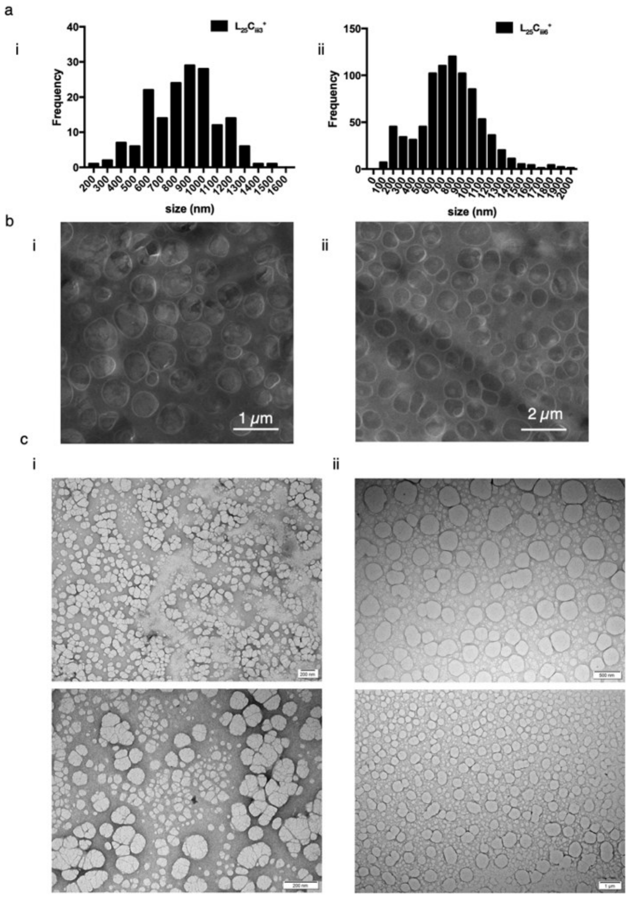

Figure S6. NPs characterisation with varying amounts of PHT. (a) The size distribution histograms plotted after measuring the size of at least 120 NPs per group from SEM micrographs for NPs prepared with 300 **(i)** and 600 µg **(ii)** of PHT. **(b)** SEM micrographs of NPs prepared with 300 **(i)** and 600 µg **(ii)** of PHT. **(c)** TEM micrographs of NPs prepared with 300 **(i)** and 600 µg **(ii)** of PHT.

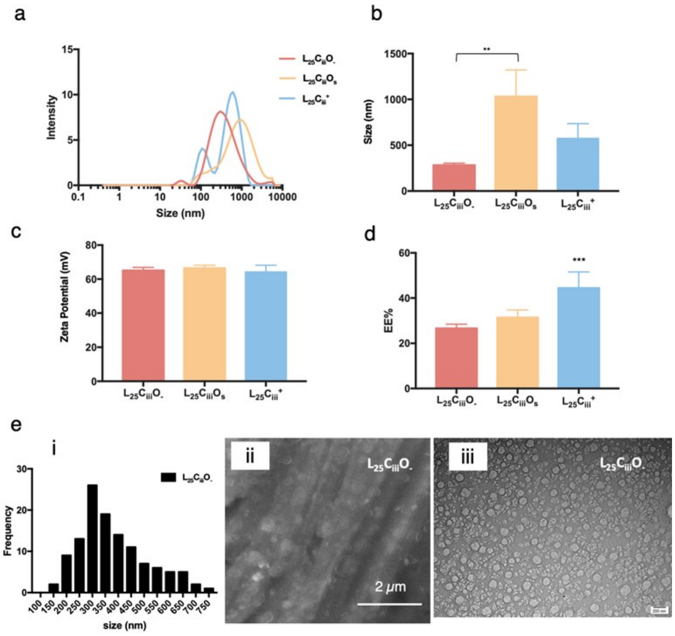

Figure S7. The effect of oil core on the properties of NPs. (a) NPs size distribution histograms and ADS of NPs were examined using DLS. Size distribution of NPs prepared without the triacetin oil core showing the smallest ADS, while the NPs size increased to 1030 nm when PHT and triacetin were added separately in the organic phase. The EE% examined using HP-TLC was significantly lower in NPs prepared without the oil core than NPs prepared with the triacetin oil core. (one-way ANOVA test; *$p < 0.05$, **$p < 0.01$, ***$p < 0.001$, n= 3 at least). **(b)** TEM micrograph of NPs prepared with the addition of PHT into NPs core without using triacetin, and with addition of PHT and triacetin separately in the organic phase.

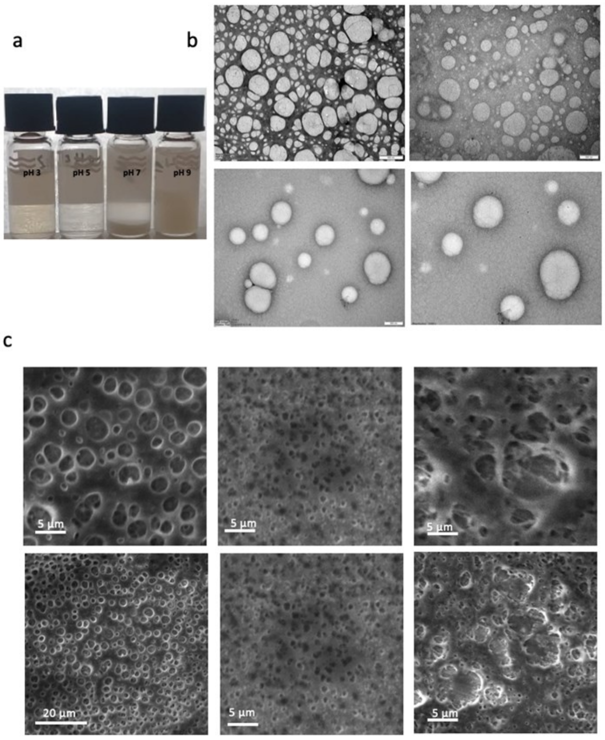

Fig. S8 (a) Visual appearance of $L_{10}C_i^+$ stored in aqueous medium with pH ranging from pH 3 to pH 9. (b) TEM micrographs showing the NPs after extended storage in solution for three months. (c) SEM micrographs of L10Ci+ stored in aqueous medium (i) pH 3, (ii) pH 7 and (iii) pH 9 at 25˚ C over one week
